# Development of a nomogram for predicting hyperkalemia in advanced chronic kidney disease

**DOI:** 10.1080/0886022X.2026.2709935

**Published:** 2026-08-04

**Authors:** Jonathan S. Chávez-Iñiguez, R. Lizzete Ornelas-Ruvalcaba, Gonzalo Rodríguez-García, Guillermo Navarro-Blackaller, Ramón Medina-González, Alejandro Martínez Gallardo-González, Luz Alcantar-Vallin, Gabriela J. Abundis-Mora, Juan A. Gómez-Fregoso, Guillermo García-García, Paula Catalina Lizarazo, Emerson Joachin Sánchez

**Affiliations:** a Nephrology Department, Hospital Civil de Guadalajara Fray Antonio Alcalde, Guadalajara, Jalisco, Mexico; b University of Guadalajara Health Sciences Center, Guadalajara, Jalisco, Mexico; c Medical Department, Astra Zeneca, Mexico City, Mexico

**Keywords:** Chronic kidney disease, hyperkalemia, nomogram, complications, kidney replacement therapy

## Abstract

Hyperkalemia (HyperK) is a potentially life-threatening complication in advanced chronic kidney disease (CKD), yet its prediction in real-world outpatient settings remains challenging. In a retrospective cohort study including 395 patients with CKD stages 4–5, all with baseline serum potassium levels within the normal range, followed for up to 2.2 years. Clinical, biochemical, and pharmacological variables were obtained from electronic health records, and logistic regression with LASSO selection was applied to identify independent predictors of HyperK. Sex-stratified nomograms were developed to facilitate individualized risk estimation, and model performance was assessed using AUROC, calibration plots, and internal validation with 1,000 bootstrap resamples. During follow-up, 303 patients (76%) developed HyperK. Independent predictors included higher serum creatinine, calcium, and age, while higher sodium levels, hemoglobin, obesity, and thiazide use were associated with lower risk. In adjusted models, men had a 49% lower risk of HyperK (OR 0.51, 95% CI 0.28–0.92). Sex-specific nomograms demonstrated good discrimination, with AUROC of 0.78 in men and 0.81 in women, and calibration analyses confirmed adequate model fit. Importantly, both models showed a high negative predictive value (>95%), supporting their use in safely identifying low-risk patients who may require less intensive monitoring. Secondary analyses showed that higher phosphate was independently associated with mortality (OR 1.74, 95% CI 1.04–2.93), while increased creatinine predicted the need for kidney replacement therapy (OR 1.29, 95% CI 1.08–1.56). These findings provide validated, sex-specific nomograms that enable individualized risk prediction of HyperK in advanced CKD, supporting personalized management in outpatient nephrology care

## Background

The kidney plays a central role in the tubular secretion of potassium [1,2]. When kidney function is impaired, the risk of hyperkalemia (HyperK) increases [3]. HyperK is defined as a serum potassium concentration >4.5 mmol/L [4]. Approximately half of all individuals with advanced chronic kidney disease (CKD) and an estimated glomerular filtration rate (eGFR) <30 mL/min/1.73 m^2^ develop HyperK [5]. Similar associations have been reported with other indicators of impaired kidney function, such as albuminuria [6]. HyperK is a common electrolyte disorder in CKD, with reported prevalence rates ranging from 10 to 50%, depending on CKD stage and concomitant use of renin–angiotensin–aldosterone system (RAAS) inhibitors. HyperK is typically classified as mild (5.5–5.9 mmol/L), moderate (6.0–6.4 mmol/L), or severe (≥6.5 mmol/L). Severe HyperK constitutes a medical emergency, as it markedly increases the risk of life-threatening cardiac arrhythmias and sudden death. Several pathophysiological mechanisms contribute to the onset of this complication during the course of CKD [7], and the risk varies by sex [8,9]. HyperK exerts a profound clinical impact [10]. Beyond its association with mortality [11], it accelerates CKD progression [12], increases healthcare costs [13], and limits the prescription of evidence-based cardio- and nephroprotective therapies [14], including RAAS inhibitors (RAASi) [15], mineralocorticoid receptor antagonists [16], neprilysin inhibitors [17], and β-blockers [18]. Once HyperK develops, treatments aimed at lowering serum potassium are initiated. The clinical dilemma lies in balancing the well-established cardiorenal benefits of these therapies against the risk of HyperK [19]. While continuation of therapy is highly desirable given their protective effects [20], this must be carefully weighed—particularly in patients with advanced CKD, where potassium binders, although effective, may not fully mitigate the risk. In such cases, the dangers of persistent or recurrent HyperK may ultimately outweigh the benefits of continued RAASi therapy. Identifying patients at increased risk of HyperK could improve clinical care and outcomes. However, predictive tools to assist clinicians in anticipating HyperK have not been extensively studied [21]. A nomogram is a graphical representation of a predictive model that enables individualized risk estimation by integrating multiple clinical variables into a user-friendly format. We therefore propose a clinically accessible nomogram to predict HyperK using routinely collected variables from patients with advanced CKD attending our clinic. To our knowledge, this is the first study to develop and internally validate a predictive nomogram specifically designed to estimate HyperK risk in patients with advanced CKD. Unlike previous studies that have primarily examined general CKD populations or hospitalized cohorts, our model incorporates readily available clinical and biochemical variables to identify high-risk individuals in the outpatient setting. This approach addresses an important gap in the current literature and provides a practical tool to support individualized decision-making in clinical nephrology.

## Methods

### Study population and data collection

We conducted a retrospective cohort study at Hospital Civil de Guadalajara Fray Antonio Alcalde, Guadalajara, Mexico, between August 2020 and June 2024. All enrolled patients were followed in the Renal Health Clinic, an interdisciplinary care model that includes the participation of a nephrology nurse, nutritionist, psychologist, and nephrologist. The clinic is designed to prevent kidney and cardiovascular disease progression, optimize the management of comorbidities, provide patient education on kidney replacement therapy (KRT), and delay CKD progression through a comprehensive, multiparametric approach. Clinical characteristics, demographic information, and laboratory values were retrospectively obtained through automated data extraction from the institutional electronic health record system. Baseline serum creatinine was defined as the most recent value available within the previous six months. Contributing factors to CKD, such as diabetes, hypertension, and chronic heart failure, were systematically identified or excluded. Laboratory data collected included hemoglobin, platelet count, leukocyte count, glucose, urea, serum creatinine, sodium, potassium, chloride, phosphate, and calcium.

The study population was restricted to patients with advanced CKD (stages 4 and 5) who were not undergoing KRT. Advanced CKD was defined, in accordance with KDIGO guidelines, as a sustained estimated glomerular filtration rate (eGFR) <30 mL/min/1.73 m^2^ for at least three months prior to study inclusion. Only patients who met this eGFR criterion were deemed eligible [22].

We analyzed patients with >3 serum potassium determinations in order to monitor their clinical course and identify HyperK. All patients had complete baseline demographic, clinical, and laboratory data, therefore, no imputation procedures were required and sample size was consistent across all regression models. The index potassium value was that obtained at the first visit. This had to be within the normal range (3.5–4.5 mEq/L). We defined HyperK as serum potassium >4.5 mmol/L, since this value is associated with mortality in CKD in different cohorts [23,24]. All candidate predictor variables, including biochemical and hematologic values, were obtained at baseline, defined as the index visit when the first serum potassium value within the normal range was recorded. These baseline values were used to predict the subsequent development of HyperK during follow-up, ensuring that all predictors were measured prior to outcome occurrence. We excluded pregnant women, individuals younger than 18 years, patients already receiving KRT, kidney transplant recipients, and those presenting with HyperK at the first visit. Major adverse kidney events (MAKEs) were defined as a composite outcome of death or initiation of KRT. All participants provided written informed consent. The study was approved by the Institutional Review Board of Hospital Civil de Guadalajara Fray Antonio Alcalde (CE 204/24). Reporting followed the Strengthening the Reporting of Observational Studies in Epidemiology (STROBE) guidelines [25] and the Transparent Reporting of a Multivariable Prediction Model for Individual Prognosis or Diagnosis (TRIPOD) statement [26].

## Objectives

The primary objective was to develop a nomogram for predicting HyperK in both sexes during follow-up, based on clinical variables collected at visits to the Renal Health Clinic. The secondary objectives were to assess the components of the MAKE composite outcome, specifically mortality and initiation of KRT during follow-up.

### Statistical methods

Normally distributed variables were assessed using the Kolmogorov–Smirnov and Shapiro–Wilk tests. Statistical analysis was performed in sequential stages to identify the most suitable predictive model. Dimensionality reduction and variable selection were performed using the least absolute shrinkage and selection operator (LASSO) regression with 10-fold cross-validation. This approach was chosen to enhance model stability and reduce the risk of overfitting, given the relatively high number of candidate predictors in relation to outcome events. Although forward logistic regression was initially explored during the preliminary analysis phase, it was not used in the final model. The definitive predictive model relied exclusively on LASSO regression, which provided superior regularization performance and more robust variable selection. The training dataset was used to construct the model with LASSO regression and 10-fold internal cross-validation. Hyperparameter selection was based on minimizing prediction error, and the final model’s performance was evaluated on a separate validation subset to prevent overfitting. No *post hoc* modifications were made based solely on training set performance.

We selected LASSO regression to reduce model dimensionality and limit overfitting, particularly given the relatively high number of candidate predictors relative to outcome events in the sex-stratified models. This regularization technique facilitated variable selection and improved model generalizability under constrained event-per-variable ratios. Prior to model development, candidate predictors were evaluated for correlation and potential collinearity. Pearson or Spearman correlation coefficients were calculated between clinically related variables, including serum sodium and diuretic use. To further assess multicollinearity, we examined pairwise correlations and calculated variance inflation factors (VIFs) for key continuous variables. Variables exhibiting high intercorrelation (|r| > 0.7) were reviewed to prevent redundancy. The LASSO method was subsequently applied to penalize and shrink correlated coefficients, thereby minimizing collinearity bias and enhancing model interpretability. Lambda selection in the LASSO regression was performed using 10-fold cross-validation, with the optimal value defined by minimizing prediction error. This process ensured adequate regularization and improved model generalizability. Model performance was evaluated using AUROC, sensitivity, specificity, and predictive values. We also assessed model calibration by generating calibration plots and calculating the Hosmer–Lemeshow goodness-of-fit test and calibration slope. We performed 1,000 bootstrap resamples to internally validate discrimination and calibration of the final models.

Differences in continuous variables between patients who developed HyperK and those who did not were compared using the Wilcoxon rank-sum test, while categorical variables were analyzed using the *χ*^2^ or Fisher’s exact test, as appropriate. Logistic regression analyses were conducted to evaluate the risk of HyperK using three different models. Model 1 was adjusted for variables with *p* < 0.1 in the between-group analysis. Model 2 applied a forward stepwise approach including all variables with *p* < 0.1. Model 3 incorporated only variables that were statistically significant in the two previous models. The dataset was randomly divided into two subsets: one for training and one for testing. The training subset was used iteratively for model development, whereas the testing subset was used only once to confirm the predictive ability of the final adjusted model. Data cleaning and analyses were performed using R, version 4.4.2.

To apply the nomogram in clinical practice, each predictor variable is located on its corresponding axis, and a vertical line is drawn to the ‘Points’ scale to determine the score attributed. The scores for all predictors are then summed to obtain a total score, which is located on the ‘Total Points’ scale. Finally, a vertical line is drawn downwards to the ‘Risk of HyperK’ scale to identify the patient’s individualized probability of developing HyperK during follow-up. This process can be readily replicated using the graphical tool, which translates multivariable regression outputs into a simple and user-friendly format.

## Results

### Description of the cohort and frequency of HyperK

**Figure 1.**
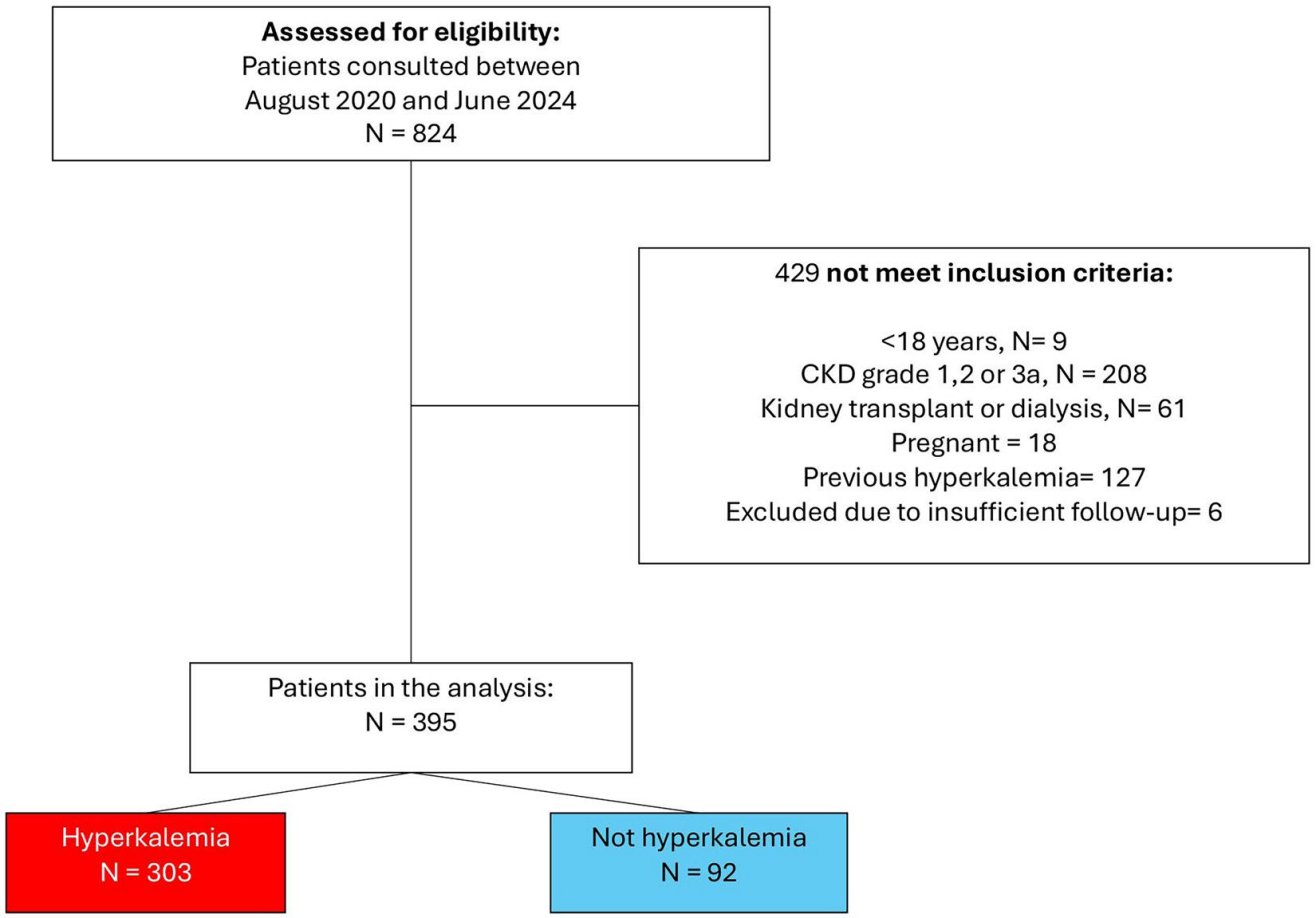
Flow chart of the study cohort.

Between August 2020 and June 2024, a total of 824 individuals attended the Renal Health Clinic. Of these, 429 did not meet the inclusion criteria, primarily because they had CKD stages 1–3 (*n* = 208) or a history of prior HyperK (*n* = 127), leaving 395 patients with advanced CKD and normal baseline serum potassium values for the final analysis. During follow-up, 303 patients (76%) developed HyperK and 92 (24%) did not, as illustrated in the flowchart (Figure 1).

Baseline characteristics according to HyperK development are presented in Table 1. The study population was representative of patients with advanced CKD: 49.4% were women, the mean age was 65 years, and the most common comorbidities were diabetes (57%) and hypertension (74.9%). The mean serum creatinine level was 2.4 mg/dL, corresponding to a mean eGFR of 25 mL/min/1.73 m^2^. At baseline, 80% of patients were receiving RAAS inhibitors and 60% diuretics. Compared with those who did not develop HyperK, patients who did had a lower prevalence of obesity (25.5 vs. 37.0%, *p* = 0.035), higher frequency of furosemide use (48.8 vs. 35.9%, *p* = 0.032), lower frequency of thiazide use (12.5 vs. 21.7%, *p* = 0.042), and lower mean calcium levels (9.1 vs. 9.2 mg/dL, *p* = 0.047). RAAS inhibitor use did not differ significantly between groups.

**Table 1.** Baseline characteristics of the entire cohort with advanced CKD according to the development of hyperkalemia during follow up.

Variable	Total (*N* = 395)	Hyperkalemia (*N* = 303)	No hyperkalemia (*N* = 92)	*P*
Female sex	195 (49.4%)	143 (47.2%)	52 (56.5%)	0.123
Age	65.0 (52.5, 74.0)	65.0 (53.0, 75.0)	61.5 (46.5, 72.0)	0.085
DM	226 (57.2%)	179 (59.1%)	47 (51.1%)	0.187
Hypertension	296 (74.9%)	232 (76.6%)	64 (69.6%)	0.216
CHF	20 (5.1%)	13 (4.3%)	7 (7.6%)	0.274
Cancer	30 (7.6%)	22 (7.3%)	8 (8.7%)	0.655
Obesity	111 (28.1%)	77 (25.4%)	34 (37.0%)	0.035
CCB	155 (39.2%)	118 (38.9%)	37 (40.2%)	0.903
RAASi	318 (80%)	268 (88.4%)	72 (78%)	0.402
Beta-blockers	71 (18.0%)	52 (17.2%)	19 (20.7%)	0.442
Furosemide	181 (45.8%)	148 (48.8%)	33 (35.9%)	0.032
Thiazides	58 (14.7%)	38 (12.5%)	20 (21.7%)	0.042
EPO	75 (19.0%)	57 (18.8%)	18 (19.6%)	0.880
sCr, mg/dL	2.4 (1.8, 3.2)	2.4 (1.8, 3.2)	2.3 (1.7, 3.3)	0.771
eGFR mL/min/1.73 m^2^	25.0 (17.5, 34.0)	25.0 (18.0, 34.0)	25.0 (16.5, 34.0)	0.530
Hemoglobin, gr/dL	12.1 (10.9, 13.3)	12.1 (11.0, 13.3)	12.1 (10.9, 13.0)	0.624
Leukocytes, µL	7.7 (6.2, 9.1)	7.7 (6.2, 9.2)	7.8 (6.3, 8.8)	0.978
Platelets, µL	232.0 (192.5, 286.0)	233.0 (192.0, 290.5)	231.0 (201.2, 276.2)	0.649
Sodium, mEq/L	137.0 (135.0, 140.0)	137.0 (135.0, 140.0)	137.5 (135.0, 140.0)	0.601
Calcium, mg/dL	9.2 (8.7, 9.5)	9.2 (8.8, 9.5)	9.1 (8.6, 9.4)	0.047
Phosphate, mg/dL	4.0 (3.5, 4.6)	4.0 (3.5, 4.6)	4.0 (3.4, 4.5)	0.555

Abbreviations: CCB, calcium channel blockers; CHF chronic heart failure; DM, diabetes mellitus; eGFR estimated glomerular filtration rate; EPO, erythropoietin; RAASi, Renin-Angiotensin-Aldosterone System Inhibitors; sCr, serum creatinine.

### Risk of HyperK during follow-up: whole cohort

Performance metrics were assessed at two classification thresholds: the default probability cutoff of 0.5 and the optimal cutoff determined by the Youden index. As expected, sensitivity and specificity varied depending on the selected threshold. At the 0.5 cutoff, the model favored specificity, whereas the Youden-based threshold provided a more balanced tradeoff between sensitivity and specificity. These findings highlight the inherent dependence of performance metrics on threshold selection and underscore the importance of clinical context when applying prediction models.

During the 2.2-year follow-up, patients had a median of four serum potassium measurements. Across the cohort, 303 patients (76%) developed HyperK and 92 (24%) did not. In the adjusted logistic regression models, the risk of developing HyperK was 49% lower in men (OR 0.51, 95% CI 0.28–0.92) and 58% lower in patients taking thiazides (OR 0.42, 95% CI 0.21–0.86). Conversely, risk increased by 2% per year of age (OR 1.02, 95% CI 1.00–1.03) and by 53% with higher serum calcium levels (OR 1.53, 95% CI 1.08–2.23), as shown in Supplementary Table 1.

The model predicted HyperK with an overall accuracy of 76.6%, sensitivity of 6.5%, and specificity of 97.9%. Thus, the model was highly specific but demonstrated low sensitivity. When the algorithm predicted that a patient would not develop HyperK, this was almost always correct, as reflected by a negative predictive value of 0.97. In contrast, when the algorithm predicted HyperK, the probability of the event actually occurring was only 6.5%. The receiver operating characteristic (ROC) curve calculated using the Youden index (Supplementary Figure 1) yielded a C statistic of 0.70, with sensitivity of 0.83 and specificity of 0.44. Therefore, we were able to construct a model with improved predictive performance, achieving a sensitivity of 83% and a specificity of 45%.

### Risk of HyperK according to sex

Given that the risk of HyperK differed by sex, we developed separate models for women and men. Table 2 presents the risk factors identified in the univariate and multivariate analyses.

**Table 2.** Factors associated with hyperkalemia in CKD patients by univariate and multivariate analyses according to sex.

	Women				Men			
	Univariate logistic regression		Multivariate logistic regression		Univariate logistic regression		Multivariate logistic regression	
Variables	OR (95% CI)	*P*	OR (95% CI)	*P*	OR (95% CI)	*P*	OR (95% CI)	*P*
Age	2.086 (0.957,4.546)	0.064	1.891(0.926,3.862)	0.081	2.507 (0.955,6.582)	0.062	2.418 (1.008,5.803)	0.048
sCr	0.705 (0.286,1.740)	0.448			0.930 (0.167,5.159)	0.933		
eGFR	1.678 (0.669,4.208)	0.270			1.467 (0.273,7.892)	0.655		
DM	0.809 (0.340,1.924)	0.632			2.929 (1.085,7.907)	0.034	2.891 (1.182,7.073)	0.020
Hypertension	2.408 (0.809,7.168)	0.114	2.242 (0.894,5.625)	0.085	1.743 (0.564,5.392)	0.335		
CHF	0.563 (0.105,3.029)	0.504			0.233 (0.045,1.206)	0.082	0.251 (0.058,1.096)	0.066
Cancer	1.077 (0.327,3.548)	0.903			0.218 (0.036,1.315)	0.097		
Obesity	0.413 (0.186,0.917)	0.030	0.409 (0.191,0.873)	0.021	0.556 (0.210,1.472)	0.237		
Hemoglobin	0.964 (0.442,2.102)	0.927			0.748 (0.309,1.812)	0.520		
Leukocytes	0.983 (0.244,3.954)	0.980			0.236 (0.060,0.929)	0.039	0.322 (0.090,1.153)	0.082
Platelets	0.807 (0.245,2.659)	0.725			0.776 (0.185,3.261)	0.729		
Sodium	0.365 (0.151,0.881)	0.025	0.396 (0.171,0.917)	0.031	1.479 (0.490,4.460)	0.487		
Calcium	1.478 (0.508,4.299)	0.473			0.422 (0.157,1.138)	0.088	0.447 (0.185,1.082)	0.074
Phosphate	0.779 (0.333,1.823)	0.564			2.203 (0.692,7.007)	0.181		
RAASi	1.119 (0.399,3.138)	0.831			0.543 (0.141,2.096)	0.376		
CCB	0.576 (0.255,1.301)	0.184	0.542 (0.250,1.174)	0.120	1.129 (0.431,2.960)	0.805		
Beta-blockers	0.737 (0.279,1.952)	0.540			0.723 (0.209,2.504)	0.609		
Furosemide	1.771 (0.800,3.920)	0.158	1.711 (0.817,3.581)	0.154	1.925 (0.762,4.862)	0.166	1.847 (0.780,4.373)	0.163
Thiazides	0.448 (0.163,1.228)	0.118	0.459 (0.175,1.199)	0.112	0.349 (0.110,1.108)	0.074	0.399 (0.139,1.150)	0.089
EPO	0.882 (0.351,2.221)	0.791			1.581 (0.418,5.978)	0.500		
Phosphate	0.782 (0.331–1.827)	0.564			2.20 (0.691–7.012)	0.181	2.458 (0.826,7.315)	0.106
Constant	1.949 (0.625,6.083)	0.250	2.214 (1.150,4.261)	0.017	2.860 (0.636,12.861)	0.171	2.095 (1.075,4.082)	0.030

All odds ratios (ORs) for continuous variables were calculated per unit increase as follows: age (per year), eGFR (per 1 mL/min/1.73m²), hemoglobin (per 1 g/dL), leukocytes and platelets (per 1,000/µL), sodium (per 1 mEq/L), calcium and phosphate (per 1 mg/dL), and serum creatinine (per 1 mg/dL). Categorical variables were coded as binary (Yes = 1 / No = 0).

Abbreviations: CCB, calcium channel blockers; CHF chronic heart failure; DM, diabetes mellitus; eGFR estimated glomerular filtration rate; EPO, erythropoietin; RAASi, Renin-Angiotensin-Aldosterone System Inhibitors; sCr, serum creatinine.

**Figure 2.**
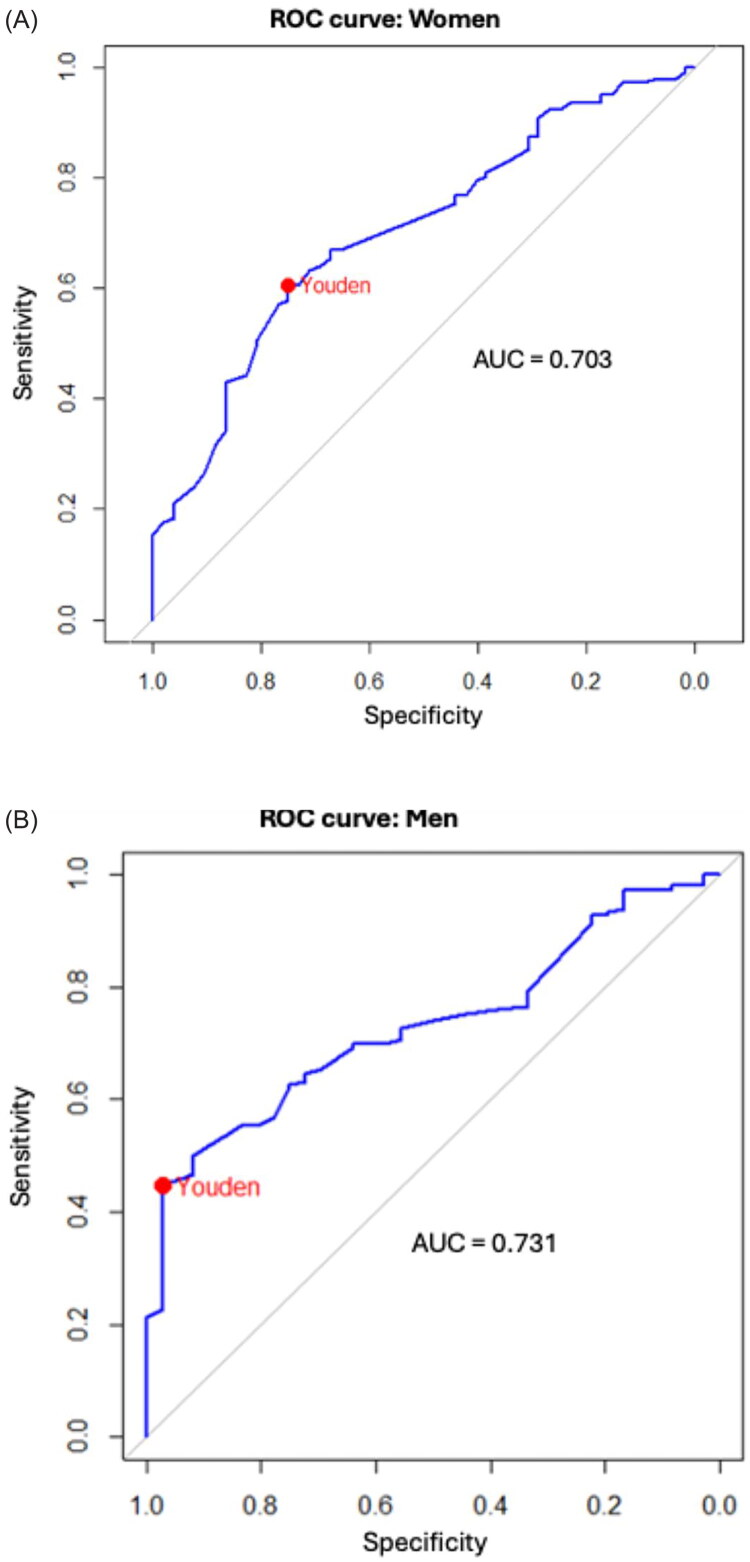
AUROC for hyperkalemia in women (A) and men (B).

In women, the adjusted analysis showed that the risk of HyperK was reduced by 60% in the presence of obesity (OR 0.40, 95% CI 0.19–0.87) and by 61% with higher sodium levels (OR 0.39, 95% CI 0.17–0.91). The ROC curve (Figure 2, panel A) yielded a Youden index of 0.356 and an AUROC of 0.703, with an accuracy of 0.742, sensitivity of 0.937, specificity of 0.211, precision of 0.764, and an F-score of 0.842.

To better interpret the model’s performance, sensitivity and specificity were reported using both the default probability threshold (used in the ROC curve) and the Youden index-derived threshold. While the default threshold yielded high sensitivity but very low specificity (6.5%), the Youden-optimized threshold provided a more balanced performance, increasing specificity while maintaining adequate sensitivity. Notably, the model demonstrated a high negative predictive value (NPV > 95%), underscoring its clinical utility in identifying patients unlikely to develop HyperK.

**Figure 3.**
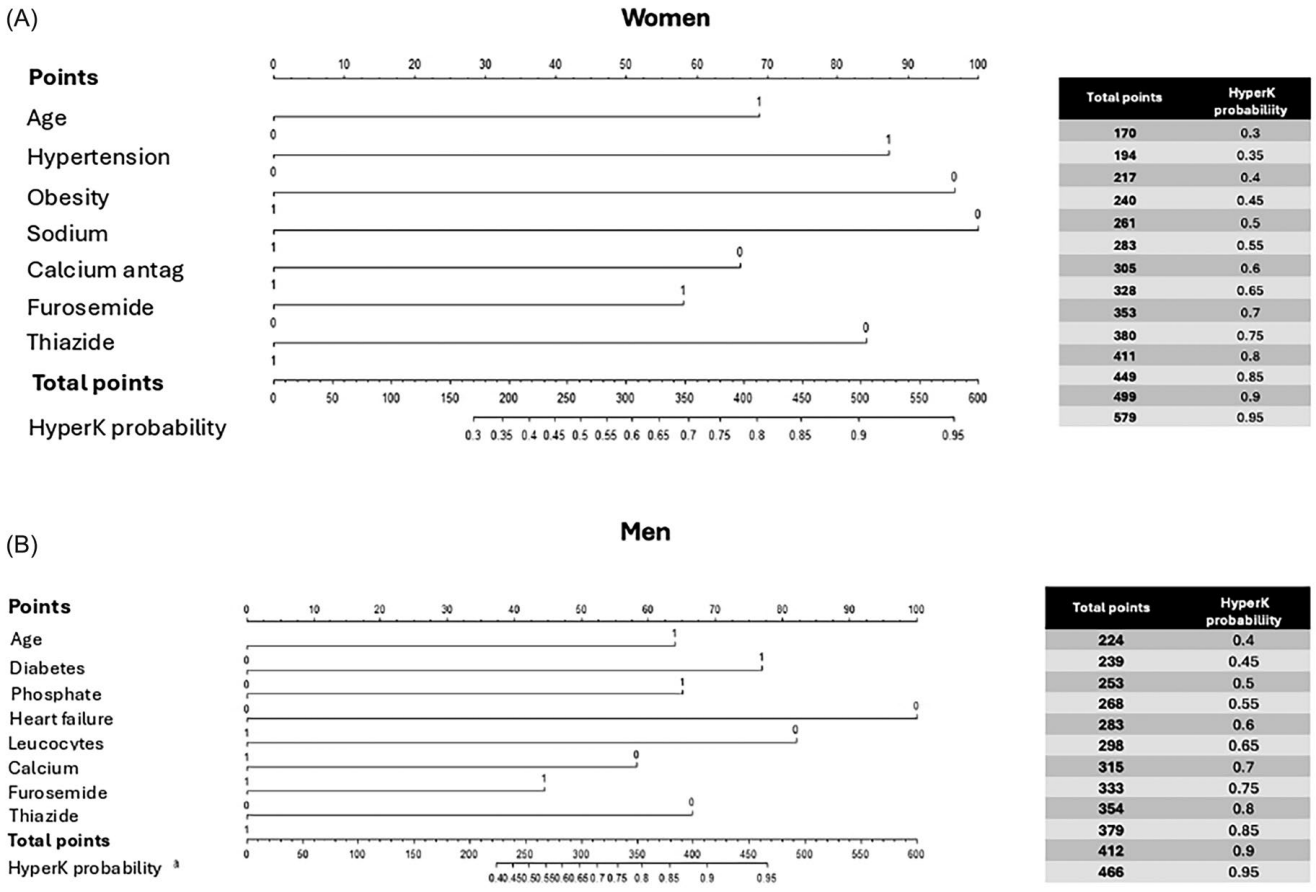
Nomogram for predicting hyperkalemia in women (A) and men (B) with advanced CKD.

We developed a nomogram to predict HyperK in women (Figure 3 Panel A).

Supplementary Table 2, panel A, presents the association between the binary values of the variables included in the predictive model and their corresponding scores in the nomogram for women. The nomogram is interpreted by summing the scores assigned to each predictor. For example, a woman aged >67 years receives 69 points; the presence of hypertension adds 87 points; absence of obesity, 95 points; low sodium, 100 points; absence of calcium antagonist use, 66 points; and furosemide use, 58 points. In this scenario, the total score would be 475 points, corresponding to a predicted risk of >80% for developing HyperK during follow-up. Comparison of predicted risk probabilities showed clear discrimination between groups (*p* < 0.001) (Supplementary Figure 2).

Using the same approach applied in women, we evaluated the risk of HyperK in men. The model demonstrated lower predictive performance in men compared with women. In the multivariate analysis, the AUROC was 0.731 and the Youden index was 0.419 (Figure 2, panel B).

Supplementary Table 2, panel B, presents the association between the binary values of the variables included in the predictive model and their corresponding scores in the nomogram for men (Figure 3, panel B). Supplementary Figure 2, panel B, illustrates the comparison of predicted HyperK risk according to the nomogram. The Kolmogorov–Smirnov test demonstrated a statistically significant difference between the two groups (*p* < 0.001).

Calibration assessment showed good agreement between predicted and observed risks. The calibration slopes were close to 1.0, and the Hosmer–Lemeshow test was not statistically significant, indicating adequate model fit. Additionally, model calibration was evaluated using calibration plots generated for the overall cohort and separately for women and men (Supplementary Figure 3). These plots demonstrated good agreement between predicted and observed risks across all groups, supporting the robustness of the model’s performance in sex-specific applications.

We increased the user-friendliness of these nomograms by means of a simple electronic application (available App Store URL: https://apps.apple.com/app/cardiorenal-calc/id6503342566 and Google PlayStore URL: https://play.google.com/store/apps/details?id=com.calculadoraderiesgos&pli=1).

### Secondary objectives: MAKEs evaluated by death and KRT

Regarding the secondary objectives, 29 patients (7%) died during follow-up. Death was more frequently associated with higher serum calcium levels (9.2 vs. 8.8 mg/dL) and lower phosphate levels (4.0 vs. 4.5 mg/dL) (both *p* < 0.05). In the adjusted analysis, phosphate was the only variable independently associated with mortality, conferring a 74% increased risk (OR 1.74, 95% CI 1.04–2.93). Kidney replacement therapy (KRT) was initiated in 73 patients (18.4%). Compared with those who did not require KRT, these patients were less likely to have cancer (0 vs. 30%), more frequently received erythropoietin (34.2% vs. 15.5%), and more often presented with hypocalcemia and hyperphosphatemia (all *p* < 0.05). In the multivariate analysis, higher serum creatinine was independently associated with a 29% increased risk of initiating KRT (OR 1.29, 95% CI 1.08–1.56).

## Discussion

In this retrospective cohort of patients with advanced CKD attending the Renal Health Clinic, 76% developed HyperK during follow-up. To predict this clinically relevant event, we developed a nomogram for individualized risk assessment based on logistic regression with LASSO, using simple and readily accessible variables. The model proved particularly useful in women. The predictive models developed in this study are intended to support physicians in clinical decision-making, including identifying patients who may require more frequent monitoring of serum potassium.

Although the AUROC values of our models (0.70–0.73) indicate moderate discrimination, they still offer practical clinical utility. In real-world nephrology practice, predictive tools with AUROC values in this range can meaningfully assist clinicians by identifying patients at particularly low or high risk, guiding the intensity of monitoring and preventive interventions. Importantly, the models demonstrated very high negative predictive value (>95%), which may help clinicians safely reduce unnecessary serum potassium measurements in low-risk individuals.

The model results should be interpreted objectively. The nomogram provides a quantitative tool to predict HyperK within comprehensive clinical assessments and can assist physicians in clinical decision-making. It is particularly useful for identifying women at high risk of HyperK and may help reduce unnecessary serum potassium testing.

Although baseline serum potassium is clinically relevant, it was not retained in the final multivariable model after LASSO regression, likely due to its transient nature and limited predictive value compared with more stable variables. The finding that male sex was associated with a lower risk of HyperK was unexpected; possible explanations include sex-related differences in hormonal regulation, dietary potassium intake, body composition, or diuretic responsiveness [7–9]. Contrary to previous reports, RAAS inhibitor use was not independently associated with increased HyperK risk in our cohort, which may reflect selection bias, whereby clinicians preferentially prescribe or adjust RAASi therapy in lower-risk patients or implement closer monitoring. Similarly, the apparent protective effect of furosemide may reflect prescription bias, as patients perceived to be at higher risk were more likely to receive loop diuretics prophylactically. Finally, although baseline eGFR was considered, it was excluded in the final model after LASSO selection, likely due to limited variability in kidney function within this advanced CKD cohort, where most patients exhibited similar degrees of impairment.

Our nomogram for predicting HyperK in advanced CKD adds to that of Xue et al. [21], although there are major differences. One of the main differences was the study populations, namely, 2 races that differ in terms of genetics and diet. However, ours differs in some important areas, mainly that of the study population, which comprised 2 separate races that differ in terms of genetics and diet. Xue et al. used a higher cutoff value to define HyperK (>5.5 mmol/L), thus accounting for the lower reported frequency of this event (27%) and the way their model was run. We preferred a lower cutoff to define HyperK (>4.5 mmol/L), because this is the value at which patients begin to be at risk of adverse events [23,24]. Serum potassium levels in advanced CKD fluctuate over time; our model accounts for this variability by estimating risk during follow-up rather than at a single point. Of note, both nomograms are fully acceptable for predicting HyperK in the populations studied.

The high frequency of HyperK observed in our cohort was expected and is consistent with previously reported data [5]. HyperK has been documented in up to 38% of patients receiving RAAS inhibitors, compared with 80% in our study population [15]. This complication carries important clinical consequences. Recent evidence shows that in patients with CKD on RAAS inhibitors who developed HyperK, hospital length of stay increased by 35% among those who discontinued therapy [20]. Similarly, data from another cohort demonstrated that 33% of patients in whom RAAS inhibitors were discontinued or dose-reduced did not restart therapy, a decision associated with increased risk of cardiorenal events [13,19].

These findings support the notion that anticipating HyperK should encourage clinicians to avoid unnecessary treatment withdrawal, thereby preventing related complications [27]. Furthermore, HyperK may be actively prevented using therapies that mitigate potassium elevation. Alongside pharmacological interventions such as potassium binders, several non-pharmacological strategies may reduce HyperK risk in advanced CKD, including dietary potassium restriction, correction of metabolic acidosis, avoidance of potassium-inducing medications (e.g. nonsteroidal anti-inflammatory drugs), and proactive management of constipation, which can impair potassium excretion. These measures should be integrated into comprehensive care plans to maintain RAAS inhibitor therapy while minimizing HyperK-related complications. Importantly, maintaining RAAS inhibitors in patients who develop HyperK has been shown to be cost-effective, provided that serum potassium levels are controlled with available measures, such as intestinal potassium binders [28].

Interestingly, RAASi use was not retained as an independent predictor of HyperK in our cohort. This finding may reflect selection and prescription bias, whereby clinicians may have preferentially maintained RAASi in patients at perceived lower risk or implemented closer monitoring strategies to mitigate HyperK in those receiving therapy. Furthermore, the high prevalence of RAASi exposure in our cohort (>80%) may have limited variability and reduced the ability to detect differences.

The MAKEs observed in this study are consistent with expectations for such a vulnerable population [29]. Seven percent of patients died during follow-up, with phosphate and calcium levels being the main associated risk factors. This finding is not unexpected given the well-established relationship between mineral and bone disorders and mortality [30]. Approximately one in five patients initiated KRT during follow-up, a frequency comparable to that reported in other regions [31]. As expected, serum creatinine, a marker of kidney function, was the variable most strongly associated with initiation of KRT.

Our findings should be interpreted in light of several limitations. First, this was a retrospective study, and multiple clinically relevant variables related to serum potassium could not be assessed, such as dietary potassium intake, urinary potassium excretion, and the use of other HyperK-associated medications. Second, we did not evaluate additional clinically relevant outcomes of HyperK, such as hospitalizations, cardiovascular events, or neuropathy. Third, sodium–glucose cotransporter 2 inhibitors (SGLT2i) were not included in the predictive model. Although emerging evidence suggests that SGLT2i reduce the risk of HyperK, their use was very limited in our cohort (<10%), primarily because patients with advanced CKD (eGFR <30 mL/min/1.73 m^2^) were not eligible for this therapy during most of the study period, and access in the public healthcare system was restricted. Consequently, inclusion of SGLT2i in the multivariable analysis was not feasible. Fourth, while the nomogram could be further enhanced by incorporating additional clinical data, future integration with artificial intelligence approaches may allow more accurate prediction of HyperK and MAKEs. Fifth, the definition of HyperK varies across guidelines and studies with some using higher cutoffs (e.g. ≥5.0 or ≥5.5 mmol/L). We selected a threshold of >4.5 mmol/L, as this value has been associated with increased mortality risk in CKD populations, applying thresholds different from those used in our nomogram could affect its performance. Sixth, no formal *a priori* power analysis was performed. As this was an exploratory study, established effect sizes for predictive modeling of HyperK were not available. Nevertheless, the number of events per variable was considered when building regression models to minimize overfitting and support model stability. Seventh, although serum bicarbonate was not available for all participants and thus excluded from the multivariable model, we acknowledge that metabolic acidosis is a recognized determinant of potassium homeostasis and may modulate hyperkalemia risk. Finally, the model was derived from a single-center retrospective dataset without external validation, which may limit generalizability. Although the model demonstrated good sensitivity, its modest specificity may increase the risk of over-alerting and unnecessary interventions. Future studies are needed to externally validate the model and optimize threshold selection based on specific clinical use cases. our findings should be interpreted as hypothesis-generating and exploratory, external validation in larger, multicenter cohorts will be necessary before our nomograms can be reliably implemented in routine clinical practice.

Seventh, the absence of a formal *a priori* power calculation; instead, we relied on events-per-variable considerations and applied LASSO regression to minimize overfitting.

The strengths of our study include the development of an online tool to simplify use of the nomogram and the fact that this is the first tool of its kind reported in Latin America, a region with a substantial burden of CKD that follows a clinical course distinct from that observed in other parts of the world [32]. Unlike traditional additive risk scores, nomograms reflect the underlying statistical model and provide continuous, individualized probability estimates, thereby enhancing clinical decision-making. Our study is clinically relevant as it demonstrated the ability to estimate HyperK risk in patients with advanced CKD with good predictive performance. The tool can be applied within seconds on a physician’s smartphone and relies on variables that are easily obtained in routine clinical practice. Assessing HyperK risk could facilitate the prescription of disease-modifying therapies, such as finerenone [33] and intestinal potassium binders [34].

## Conclusions

We developed a nomogram based on simple clinical characteristics to estimate the risk of HyperK in patients with advanced CKD. The tool demonstrated good predictive accuracy and enables quantitative identification of individuals at high risk. Furthermore, a web-based version of the nomogram may facilitate comprehensive clinical assessment and support decision-making in routine practice.

## Supplementary Material

Supplemental material 2_12_25.docx

## Data Availability

The data that support the findings of this study are available from the corresponding author (JSCI), upon reasonable request.

## Abbreviations

AUROC: area under the ROC curve
CHF: congestive heart failure
CKD: chronic kidney disease
CKD-EPI: CKD Epidemiology Collaboration
DM: diabetes mellitus
eGFR: estimated glomerular filtration rate
EPO: erythropoietin
HyperK: hyperkalemia
KRT: kidney replacement therapy
KDIGO: Kidney Disease: Improving Global Outcomes
lasso: least absolute shrinkage and selection operator
MAKE: major adverse kidney event
OR: odds ratio
RAASi: renin-angiotensin-aldosterone system inhibitor
ROC: receiver operating characteristic
sCr: serum creatinine
STROBE: Strengthening the Reporting of Observational Studies in Epidemiology
TRIPOD: Transparent reporting of a multivariable prediction model for individual prognosis or diagnosis
