# Supplementary material for: Development of a nomogram for predicting hyperkalemia in advanced chronic kidney disease
Source: Ren Fail. 2026 Aug 4;48(1):2709935. doi: 10.1080/0886022X.2026.2709935 (PMC13455697; doi:10.1080/0886022X.2026.2709935)
Supplement: Supplementary file 1 [file IRNF_A_2709935_SM3588.docx]

**Supplemental material**

**Supplemental Table 1.** Adjusted odds ratios (ORs) obtained from the binary logistic regression model.

| **Variable** | **OR** | **CI** |
| --- | --- | --- |
| intercept | 0.04 | 0.00-0.97 |
| Sex | 0.51 | 0.28-0.92 |
| Age | 1.02 | 1.00-1.03 |
| Calcium | 1.53 | 1.08-2.23 |
| Thiazide | 0.42 | 0.21-0.86 |

Supplemental Figure 1. Receiver operating characteristic (ROC) curve for the hyperkalemia predictive model.

**Supplementary Table 2.**

Binary values of the variables included in the predictive model and their corresponding scores in the nomogram for women (A) and men (B).

**A) Women**

| **Variable** | **Bit** | **Points** |
| --- | --- | --- |
| Age | 0 | 0 |
|  | 1 | 69 |
| Hypertension (HTN) | 0 | 0 |
|  | 1 | 87 |
| Obesity | 0 | 97 |
|  | 1 | 0 |
| Sodium | 0 | 100 |
|  | 1 | 0 |
| Calcium channel blocker use | 0 | 66 |
|  | 1 | 0 |
| Furosemide use | 0 | 0 |
|  | 1 | 58 |
| Thiazide use | 0 | 84 |
|  | 1 | 0 |

**B) Men**

| **Variable** | **Bit** | **Points** |
| --- | --- | --- |
| Age | 0 | 0 |
|  | 1 | 64 |
| Diabetes mellitus (DM) | 0 | 0 |
|  | 1 | 77 |
| Congestive heart failure (CHF) | 0 | 100 |
|  | 1 | 0 |
| Leukocytes | 0 | 82 |
|  | 1 | 0 |
| Phosphate | 0 | 0 |
|  | 1 | 65 |
| Calcium | 0 | 58 |
|  | 1 | 0 |
| Furosemide use | 0 | 0 |
|  | 1 | 44 |
| Thiazide use | 0 | 66 |
|  | 1 | 0 |

**Supplemental Figure 2**. Discrimination between groups for the development of hyperkalemia in women (**A)** and men **(B).**

A) Women

B) Men

**Supplemental Figure 3**. Calibration plots for the entire cohort, for females, and for males.
